# A framework for understanding an open scientific community using automated harvesting of public artifacts

**DOI:** 10.1093/jamiaopen/ooae017

**Published:** 2024-02-29

**Authors:** Star Liu, Asieh Golozar, Nathan Buesgens, Jody-Ann McLeggon, Adam Black, Paul Nagy

**Affiliations:** Biomedical Informatics and Data Science, Johns Hopkins University School of Medicine, Baltimore, MD 21205, United States; OHDSI Center at the Roux Institute, Northeastern University, Boston, MA 04101, United States; Odysseus Data Services, Cambridge, MA 02142, United States; Biomedical Informatics and Data Science, Johns Hopkins University School of Medicine, Baltimore, MD 21205, United States; Biomedical Informatics, Columbia University, New York, NY 10032, United States; Odysseus Data Services, Cambridge, MA 02142, United States; Biomedical Informatics and Data Science, Johns Hopkins University School of Medicine, Baltimore, MD 21205, United States

**Keywords:** information management, information storage and retrieval, software, web browser

## Abstract

**Background:**

The Observational Health Data Sciences and Informatics (OHDSI) community has emerged as a leader in observational research on real-world clinical data for promoting evidence for healthcare and decision-making. The community has seen rapid growth in publications, citations, and the number of authors. Components of its successful uptake have been attributed to an open science and collaborative culture for research and development. Investigating the adoption of OHDSI as a field of study provides an opportunity to understand how communities embrace new ideas, onboard new members, and enhance their impact.

**Objective:**

To track, study, and evaluate an open scientific community’s growth and impact.

**Method:**

We present a modern architecture leveraging open application programming interfaces to capture publicly available data (PubMed, YouTube, and EHDEN) on open science activities (publication, teaching, and engagement).

**Results:**

Three interactive dashboard were implemented for each publicly available artifact (PubMed, YouTube, and EHDEN). Each dashboard provides longitudinal summary analysis and has a searchable table, which differs in the available features related to each public artifact.

**Conclusion:**

We discuss the insights enabled by our approach to monitor the growth and impact of the OHDSI community by capturing artifacts of learning, teaching, and creation. We share the implications for different users based on their functional needs. As other scientific networks adopt open-source frameworks, our framework serves as a model for tracking the growth of their community, driving the perception of their development, engaging their members, and attaining higher impact.

## Background

The Observational Health Data Sciences and Informatics (OHDSI) community was founded with the mission to promote better evidence for healthcare and medical decision-making.^1^^,^^2^ Since 2014, the OHDSI community has continued to expand its network and body of literature in data standards, data characterization, safety surveillance, treatment effectiveness, risk prediction, and quality improvement. OHDSI has attracted over 2000 unique published researchers across institutions and accumulated 500 manuscripts indexed on PubMed.

As an open science community, OHDSI is committed to transparency in developing evidence. This commitment has led to over 800 videos being uploaded to the official OHDSI YouTube channel since 2016. These videos are recordings from conferences, tutorials, workshops, as well as working group meetings deemed of educational value. Since 2014, those videos have been watched for a cumulative of over 264 000 h. With the support of the European Health Data Evidence Network (EHDEN),^3^ EHDEN Academy,^4^ an open-source Moodle-based learning management system, was deployed as OHDSI’s free online learning center. To date, over 25 courses have been released on this platform with over 3400 course completions.

As the network has evolved into a unique scientific community, there is a need for continuous evaluation of the community for aligning prospective research efforts. To measure the uptake of new ideas and members and how they change over time, we need metrics that are identifiable and trackable.

We developed a software system to track community activities through automated approaches to better understand how the community is onboarded and engaged in scientific activities. This approach provides a novel way to track open science communities’ impact and productivity using publicly available artifacts. While the list of publicly available artifacts is not comprehensive, it nonetheless provides a foundation to begin to understand the health of a community. The system itself is available as an open-source project on GitHub for any open science communities: OHDSI/Community Dashboard: Dashboard to monitor the health of the OHDSI community. With the open-source framework (Community Dashboard: https://dash.ohdsi.org), we seek to achieve and answer the following:

1. Evaluate the capture and automatic tracking of evidence of OHDSI’s training, engagement, and impact through public application programming interfaces (APIs).
2. Understand the process of onboarding into the community as well as highlighting important activities via the creation of an interactive interface.
3. Provide users with easier ways to find content and become more involved in activities.

## Methods and implementation

The proposed framework currently has 3 components matching the major areas of activities (scholarly publications, teaching, and training resources): (1) PubMed, (2) YouTube, and (3) EHDEN Academy online training resources. Each of the components leveraged existing public artifacts, API services, and automated pipelines for information retrieval and aggregation. Finally, they were aggregated on a single platform, serving as a central repository of OHDSI-related artifacts and as a monitoring system for growth.

### PubMed

**Figure 1.**
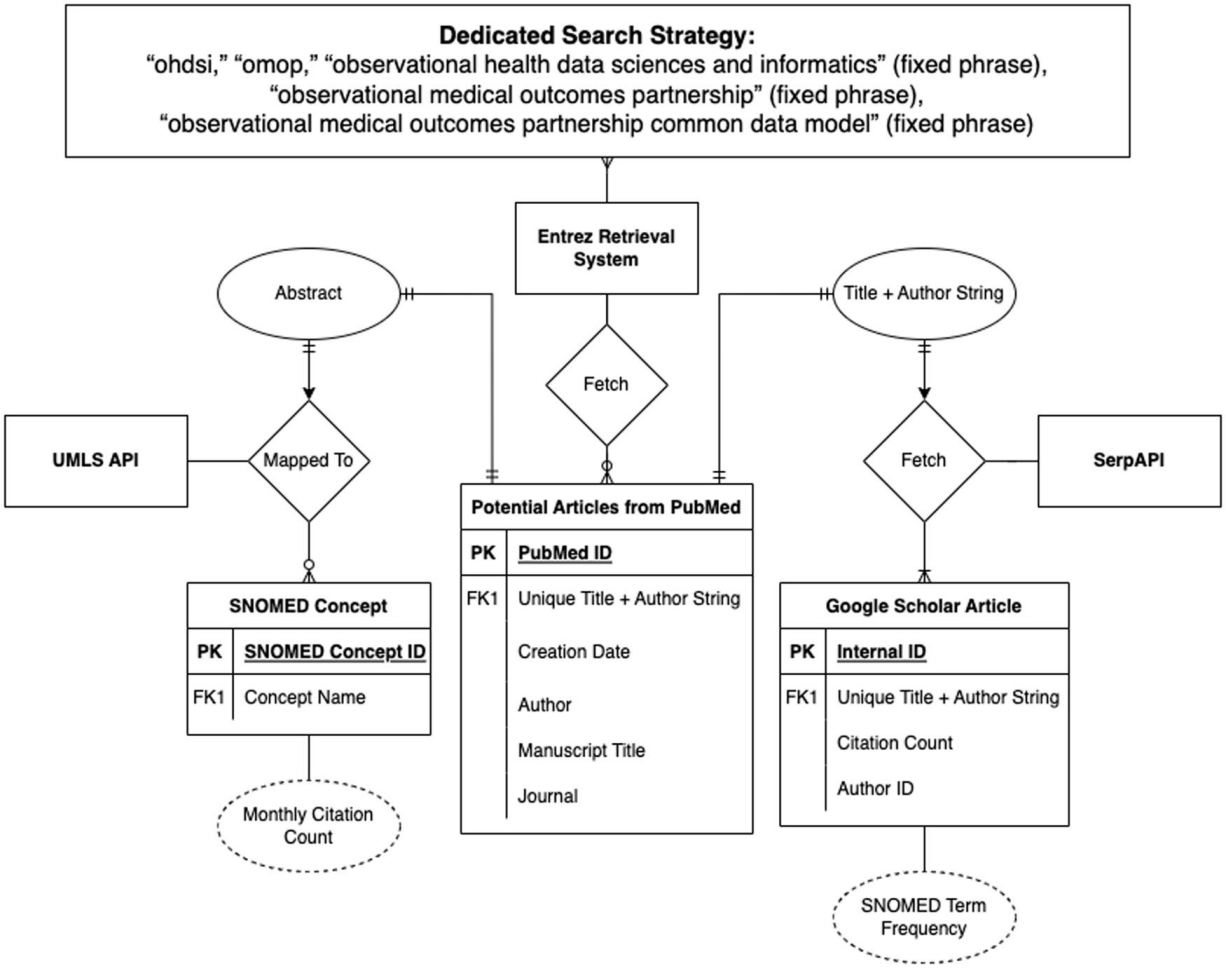
Publication entity relationship diagram.

The PubMed article entity was the central artifact in which the rest of the attributes were built as shown in the entity-relationship diagram (Figure 1). PubMed is the standard source for publication in the medical community.

The United States National Library of Medicine offers an open, free API service through its Entrez information retrieval system.^5^ This service gives access to the PubMed database, where all articles were drawn. We validated a manual tracking effort based on keyword combinations. The goal was to accurately identify OHDSI articles with high specificity. As such, our search strategy on PubMed included the search for terms “ohdsi,” “omop,” “observational health data sciences and informatics” (fixed phrase), “observational medical outcomes partnership” (fixed phrase), and “observational medical outcomes partnership common data model” (fixed phrase). The search strategies were used to fetch articles with matching strings in the title, author, or abstract. Finally, we created manual addition capabilities for edge cases. Each article from the PubMed database carried the following set of metadata: title, authors, date, journal name, Medical Subject Headings (MeSH) terms, and grant funding.

Although PubMed articles came with a set of MeSH terms, we wanted to capture the comprehensive list of clinical domains in SNOMED which is a standard vocabulary within OHDSI. We employed a 2-step process to achieve this goal. The *en_ner_bc5cdr_md* NER model (from SciSpacy) was used to extract Unified Medical Language System (UMLS) entities from the abstract,^6–8^ and the UMLS API was used to map UMLS entities to standardized SNOMED CT (US Edition) terminologies. We derived the frequencies at which the SNOMED terms appear in the abstract to assess relative importance.

While PubMed hosts all the relevant articles, it does not monitor citation count, which is a measure of scholarly dissemination and impact. Google Scholar’s algorithm tracks articles’ citation counts, and we leveraged Google Search Engine Application Program Interface (SerpAPI) to link citation counts to each PubMed article.^9^ To find the citation count, we find the corresponding article on Google Scholar. Our search strategy on Google Scholar was a combination of the article title and the first author’s name as a single unique string. Additionally, we leveraged the Levenshtein fuzzy matching algorithm (scoring cutoff = 0.95) to exclude possible edge cases where the search could be one-to-many.^10^ Only the article with the highest Levenshtein fuzzy matching score was retrieved from Google Scholars and its citation count was added to the primary PubMed articles table. Using the same Levenshtein matching algorithm, this tool identified the number and the rate of new members.

### YouTube

**Figure 2.**
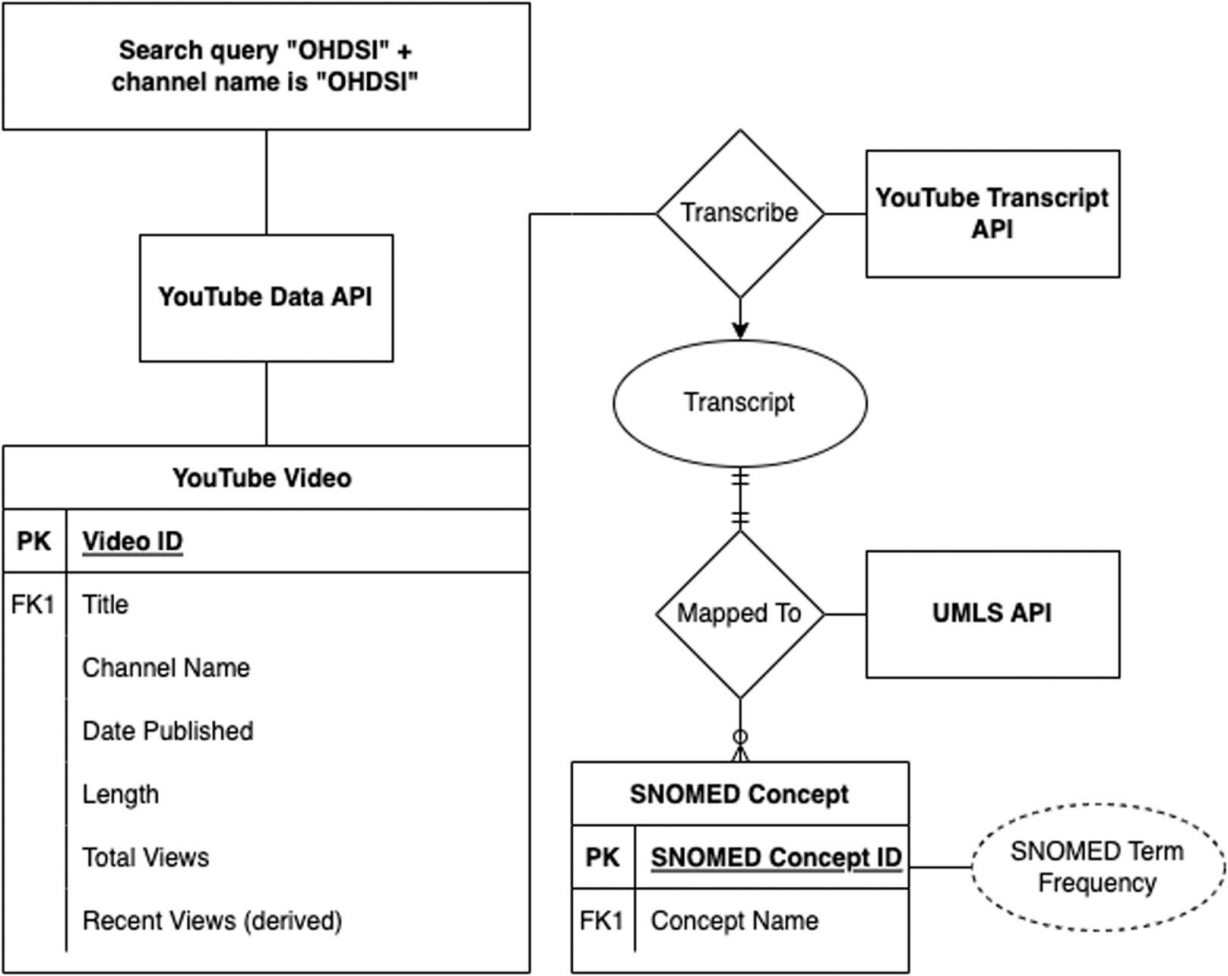
Video entity relationship diagram.

The YouTube video entity was the center around which the rest of the video attributes were built as shown in the entity-relationship diagram (Figure 2). The YouTube Data API provides aggregate user counts, and no YouTube viewer identifiers were acquired via this method.^11^ The search strategy for OHDSI-related videos involved a query for “OHDSI” in the video title and as the channel name. Videos fetched from the API carry the following metadata: video name, channel name, data, and viewership statistics.

To assess the video content characteristics, we used the free YouTube Transcript API service for automated transcript generation.^12^ Transcripts served as the textual basis from which we categorize the video content. We employed a 2-step process to achieve this goal. The en_ner_bc5cdr_md NER model (from SciSpacy) was used to extract UMLS entities from the video transcripts, and the UMLS API was used to map UMLS entities to standardized SNOMED terminologies. Using string-matching techniques, we derived the frequencies at which the SNOMED terms appear in the videos to assess relative importance.

### EHDEN

EHDEN Academy hosts a data API service that gave us access to metadata on the type of course offering and completion rates. As part of the maturity model, the EHDEN dashboard allowed us to track the level of education and training resources published and engagement for the OHDSI community. The API was designed to only provide aggregate user counts and no learner-identifiable information was shared.

The PubMed, YouTube, and EHDEN Academy data API connections were programmed in Python Version 3.7.^13^ Data updates and retrievals ran automatically and daily as Azure Functions on the Azure Cloud.^14^ Each article or video was stored as a single JSON document, and the final collection of articles and videos was stored on Azure CosmosDB. The final dashboard was built upon the Flask App framework in the backend and HTML and Dash Apps in the front end.^15^^,^^16^ All code has been published online, and we used GitHub for version control.

## Results

Summary statistics were presented as a combination of line and bar charts to provide analysis over time. Each dashboard also had a searchable table, which differed in the available features associated with each public artifact. Additionally, the search field for each feature allowed for on-demand details and filtering. The search and sort fields allow researchers to quickly identify new or highly cited resources.

**Figure 3.**
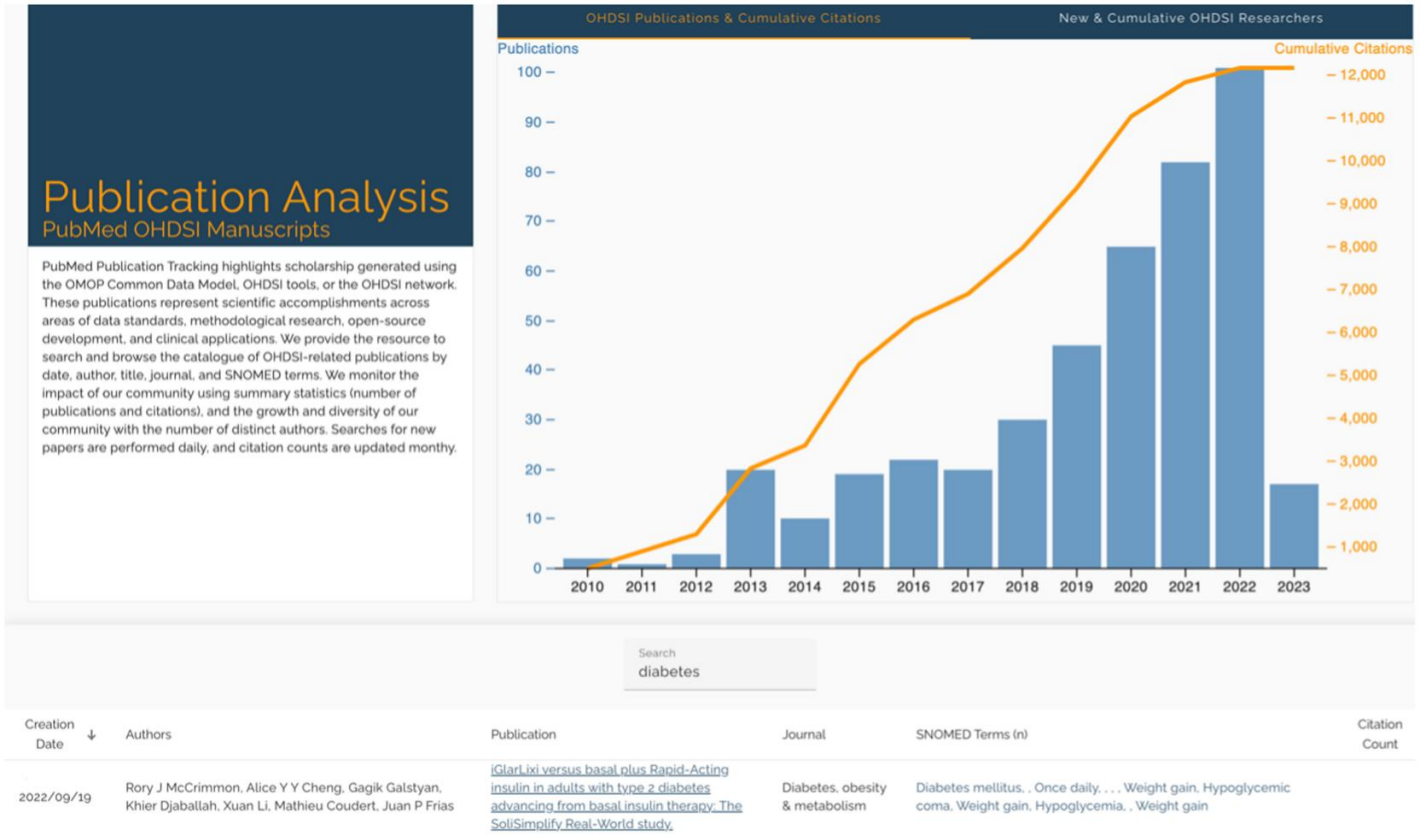
Publication dashboard showing summary figures on citations and authorship as well as a searchable table by article ID, date, authors, title, journal, content, and citation counts.

The publication dashboard tracks scholarship generated using the observational medical outcomes partnership (OMOP) Common Data Model (CDM), OHDSI tools, or the OHDSI network (Figure 3). To demonstrate trends in scholarly impact, incremental and cumulative growth was displayed for authors, published work, and citations.

The key use cases for this view were as follows:

1. Help a researcher stay up-to-date by sorting new articles recently published.
2. Help a researcher find relevant articles for citation in manuscript creation through a search text field for title, journal, author, and SNOMED terminology.
3. Help a researcher with grant writing by providing a highly cited articles sort function.
4. Provide the community with a view of overall growth in time in the number of articles as well as the number of new authors in our field.

**Figure 4.**
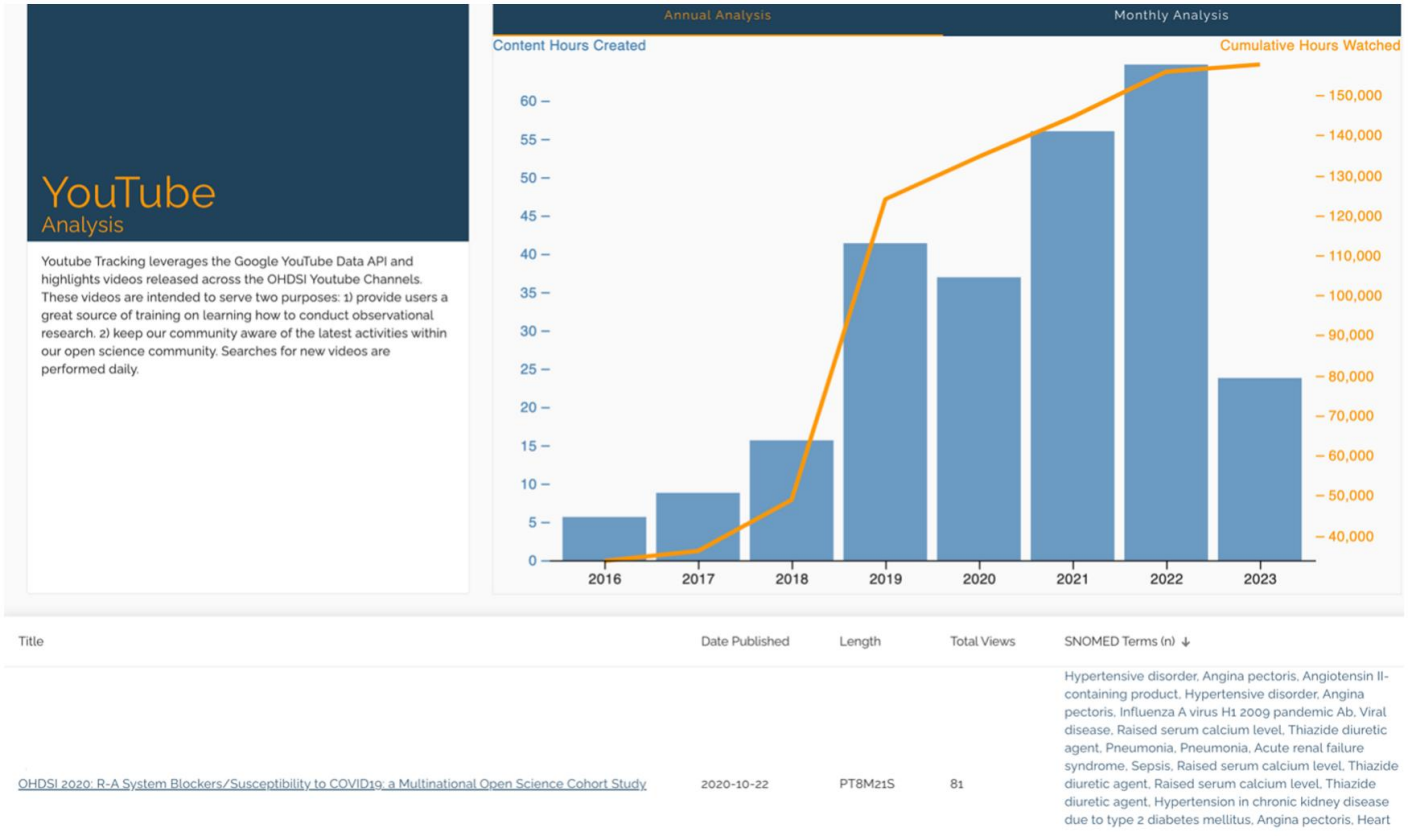
YouTube dashboard showing summary figures on historic and recent viewership as well as a searchable table by video title, date, duration, journal, content, and citation counts.

The YouTube dashboard tracks all OHDSI-related videos, including working group meetings, and community meetings (Figure 4). Like the publication dashboard, we showcased incremental and cumulative viewership in a combination of line and bar charts. We also provided the same ability to search and browse the catalog of OHDSI-related videos by date, title, duration, and SNOMED terms associated with each video.

The key use cases for this view were as follows:

1. Help a researcher stay up-to-date by sorting new videos recently published.
2. Help a researcher find the most viewed video around a topic.
3. Help a researcher the ability to search by title and SNOMED terminology.
4. Provide the community with a view of overall growth in the number of videos created as well as the cumulative number of hours watched.
5. Help educators flag videos for content that needs to be updated by looking for dated videos still being heavily viewed by the community.

**Figure 5.**
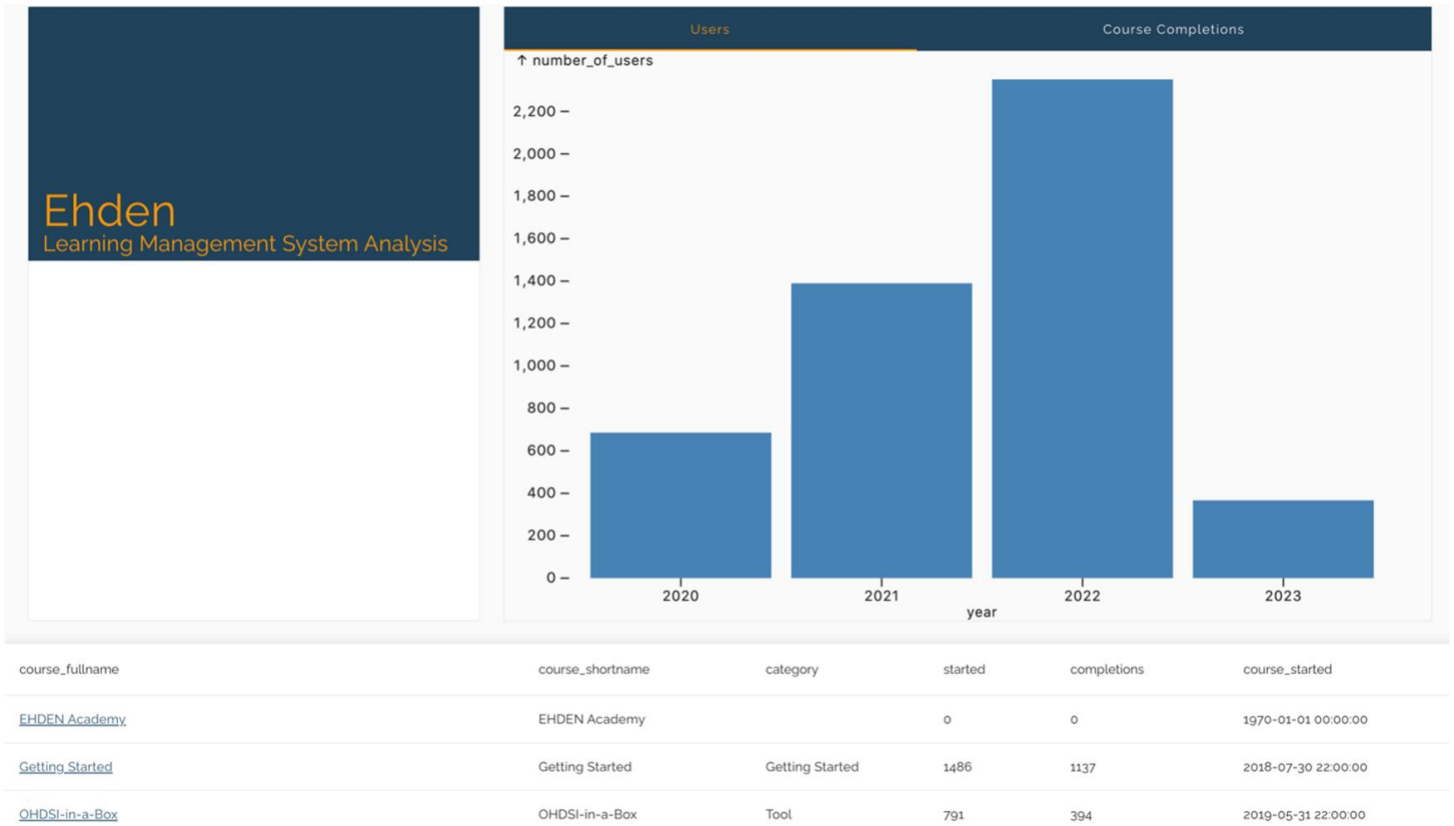
EHDEN dashboard shows summary figures on users and course completions as well as a searchable table by course name, category, date, and course author.

The EHDEN dashboard tracks all OHDSI courses published on the EHDEN Academy, broken down by the type of courses (tool, skill, methods, etc.) (Figure 5). The summary measures for this dashboard included 2 bar charts showing cumulative new learners and course completions on EHDEN Academy. We also provided the same ability to search and browse the catalog of courses by title, category, date, and course author.

The key use cases for this view were as follows:

1. Help a researcher stay up-to-date by sorting out new courses recently published.
2. Help a researcher find the most taken courses.
3. Help a researcher the ability to search by title and author to find their next course to take.
4. Provide the community with a view of overall growth in the time of course created as well as the number of learners that have completed courses by year.
5. Provide educators feedback on course updates and completion rates.
6. Help educators flag courses for content that needs to be updated by looking for dated courses still being viewed by the community.

## Discussion

We developed an open-source framework to evaluate the health and impact of an open-science scientific community. Our approach studies the OHDSI community’s major areas of activities: scholarly publications, teaching, and training resources. We built dedicated dashboards tracking each public artifact (PubMed, YouTube, and EHDEN). Our framework could serve as a basis for other open science communities to understand their network, track their growth, and enhance their impact.

Publication and citation tracking serve as evidence of impact. Over the last decade, the OHDSI network has accumulated over 12 000 citations on over 500 articles and maintained a steady growth (+1200 citations per year). Additionally, the network has seen growth in the number of new authors over the years and attracted 2000 coauthors globally by December 2022. Over 94% (477/507) of published papers resulted from the collaboration of 3 or more coauthors and most publications concentrated among a smaller group of researchers. This finding is likely the result of early-stage researchers participating in research led by senior and more tenured authors. As overall tenure increases, we expect to see a diffusion of creation as more researchers lead original research and network studies.

We also see evidence of learning. Over 800 videos have been published by OHDSI-related channels worldwide and have accumulated over 208 000 h of content watched. Videos have been published every week, including community calls, symposium talks, workshops, and tutorials. Not only is this a medium for research marketing and dissemination, but it is also a platform for education and learning. At EHDEN Academy, 20 authors altogether have built 15 courses, and over 4000 users have completed more than 3000 courses. Of the 15 courses, 6 focus on OHDSI tools and software, 6 focus on data analytic skills, and 1 focus on the methods for health technology assessment.

Tracking the areas of activities allows us to identify contributions that get rewarded for promotion and career development. The review, promotion, and tenure (RPT) practices have been studied for decades and yet they have gradually increased the emphasis on research at the expense of other means.^17–19^ Studies in the past have raised concerns regarding the inability of the current reward system to keep up with evolving technology and research practices.^20^^,^^21^ Simultaneously, approaches to evaluating RPT efforts and processes have been limited and lacked coherence with capturing activities beyond publications.^20^^,^^22^ Tracking on an individual level would maximize the value of monitoring growth and impact, but we decided to track on a community level due to privacy considerations. The OHDSI community has produced trackable and measurable artifacts around research, teaching, and services, hence offering a unique environment to study its reflection of traditional RPT processes.

Strategically, this tool provides a solution for ongoing monitoring of the community’s academic productivity, research dissemination, and growth. In contrast to manual curation and monitoring of OHDSI-related production, the publication dashboard offers a simple solution for tracking academic production and efforts. The tool can also identify, support, and recognize key contributors, who then can translate lessons learned to the rest of the community and early-stage researchers. As new researchers continue to join and OHDSI continues to expand its suite of solutions, its members’ expertise and needs shift. The YouTube and EHDEN dashboards help distill which educational content is most sought after by its members and create additional content tailored to the community’s interests. In aggregate, this tool facilitates the understanding of how the pieces of the community are interconnected, how to manage its resources, and how to maximize impact both internally and externally.

From an individual OHDSI researcher’s view, our tool facilitates learning, research, and career development. Currently, researchers need to develop a high-sensitivity search strategy on PubMed to find OHDSI studies, and the publication dashboard automates the manual search process. The catalog of articles allows researchers to learn about past OHDSI studies, and the SNOMED search capability allows for further filtering based on the clinical domain. Researchers with a clinical background can learn how OHDSI may be applied to their clinical domains and learn from other studies. Researchers with a technical background can learn about research topics that could benefit from their skillset. On the other hand, the YouTube and EHDEN dashboards allow researchers to navigate and pinpoint the types of working groups and training resources that they need to conduct their studies. Furthermore, the publication dashboard can be leveraged for finding reference material, conducting literature reviews, and writing. Researchers can also use this tool to find past funding opportunities that have supported OHDSI studies and use those as references in future grant writing. Lastly, in identifying reference studies and relevant grants, researchers can find potential mentors and collaborators both for their studies and career development.

The proposed framework enables the automatic tracking of artifacts through APIs, thereby allowing a scientific community to understand what it takes for the field to succeed. Evaluating the growth of a community as individuals move through different stages of engagement along their journey provides ongoing support for learning, participation, and research creation. Tracking the areas of activities also enables the community to provide platforms and opportunities for researchers to engage in activities recognized for promotion and career development.

### Ongoing and future work

There are limitations to our current approach. First, most of the literature, videos, and training resources on this dashboard were in English. SNOMED terminology mapping on both the publication and YouTube dashboards also excluded other languages. Second, we restricted publications to those indexed in PubMed. Future work should consider capturing OHDSI studies from other sources. Third, we did not quantitatively validate SNOMED mapping accuracy in this study. Future studies could assess mapping accuracy (ie, *F* score) and provide more precise tagging of the artifacts. Fourth, using automated transcripts for NER needs to be further refined to minimize noise and maximize its value in aiding the search for information, concepts, and knowledge. Fifth, future studies should also assess more complex growth and change over time, such as the diversity of authors, institutions, and clusters of coauthors/contributors. Lastly, GitHub analytics is an area of future activity to study engagement with software development.

As more scientific communities leverage open scientific processes, they can adopt similar frameworks underlying the OHDSI Community Dashboard, which leverages existing and growing public artifacts, to systematically monitor their growth. Beyond publications and citations, our framework accounts for other forms of societal and scientific contributions that any scientific community should care about. Ultimately, our framework and approach serve as a model for all open-source scientific communities and initiatives to drive the perception of their development and achieve higher impact.

## Conclusion

The proposed framework has been deployed as an open-source interface with interactive dashboards and data tables. Using the OHDSI community as a case study, we discuss the insights enabled by this approach and the implications for its users based on functional needs. As other scientific communities transition to open source, our framework serves as a model for driving the perception of their development, supporting their members, and achieving higher impact.

## Authors contributions

S.L. drafted the final manuscript. S.L., N.B., A.B., and P.N. conducted the implementation of the method. All authors contributed to the design and analysis. This manuscript has been read and approved by all the authors involved.

## Data Availability

All derived data are available at: https://github.com/OHDSI/CommunityDashboard/tree/main/test/exports. Raw data used in this study were accessible through several Application Programming Interfaces (APIs). PubMed publication data were obtained using the Entrez API offered by the United States National Library of Medicine. Documentation is available at: https://www.ncbi.nlm.nih.gov/books/NBK25501. Documentation on the Google YouTube Data API is available at: https://developers.google.com/youtube/v3. Documentation on the YouTube Transcript API is available at: https://github.com/jdepoix/youtube-transcript-api. The European Health Data Evidence Network (EHDEN) Academy data on its training courses were available through a custom API offered by EHDEN. Additional data will be shared on reasonable request to the corresponding author.
